# Hypopyon after Primary Cryopreserved Amniotic Membrane Transplantation for Sterile Corneal Ulceration: A Case Report and Review of the Literature

**DOI:** 10.1155/2021/9982354

**Published:** 2021-06-15

**Authors:** Kostas G. Boboridis, Dimitrios G. Mikropoulos, Nick S. Georgiadis

**Affiliations:** 3rd Ophthalmology Department, Aristotle University of Thessaloniki, Kiriakidi 1, Thessaloniki 546 21, Greece

## Abstract

*Purpose*. To report the acute development of hypopyon after primary cryopreserved amniotic membrane transplantation (AMT) for persistent corneal epithelial defect and sterile ulceration. *Case Presentation*. A selected case report of a 71-year-old male who underwent primary cryopreserved AMT for the management of long-standing corneal epithelial defects and stroma thinning. The patient developed 2 mm sterile hypopyon within 48 hours after AMT for corneal surface reconstruction. He responded well to the intensified routine postoperative topical treatment of steroid and antibiotic eye drops with the hypopyon resolving completely one week later. Five weeks after surgery, the corneal surface was smooth and epithelialized with no anterior chamber reaction or recurrence of hypopyon. *Discussion*. Hypopyon may develop as a rare complication of primary cryopreserved AMT for sterile corneal defects. It may be attributed to immunologic or hypersensitivity reaction and should be differentiated from active ocular infection as it resolves spontaneously with the routine postoperative topical treatment of steroid and antibiotic drops.

## 1. Introduction

Amniotic membrane transplantation (AMT) has been extensively used for ocular surface reconstruction with a wide range of indications. It provides a biological scaffolding which exhibits distinct antiadhesive, bacteriostatic, and epithelializing properties. The amniotic membrane (AM) and its epithelial cells do not express human leukocyte antigens; it is therefore believed to be immunologically inert and does not initiate an immunologic response of the recipient [1]. AM has been transplanted on to the ocular surface as a patch or a graft for persistent corneal epithelial defects, conjunctival defects, symblepharon, pterygia, chemical burns, and bullous keratopathy [2].

The purpose of this report is to present the acute development of sterile hypopyon into the anterior chamber following primary cryopreserved AMT onto the corneal surface for persistent sterile corneal epithelium defect and stromal thinning. A written informed consent for anonymized patient information and images to be published was provided by the patient.

## 2. Case Presentation

A 71-year-old male patient with a persistent central corneal epithelial defect and stromal thinning of 5 mm diameter on his right eye with no evidence of infection or corneal neuropathy was intermittently responsive to conservative topical treatment with antibiotic drops and ointment, intensive lubricating drops, and bandage contact lenses. The central cornea had a thinner area of 2 mm diameter with only 1/3 of the stroma remaining before Descemet's membrane and showed no sign of healing with only intermittent epithelialization despite the conservative treatment for over eight weeks from presentation. There was no evidence of corneal hypesthesia, and repeated corneal swabs for microbiology investigation proved the sterility of the lesion. The clinical suspicion of corneal rubbing against the pillow because of his sleeping preference on his right side was the most probable cause of his condition.

As counselling for his sleep pattern and conservative lubricating treatment had no effect, we proceeded with surgical management of his corneal defect using cryopreserved AM. We followed our previously described standard technique for corneal surface reconstruction with the membrane placed over the whole cornea [2].

**Figure 1:**
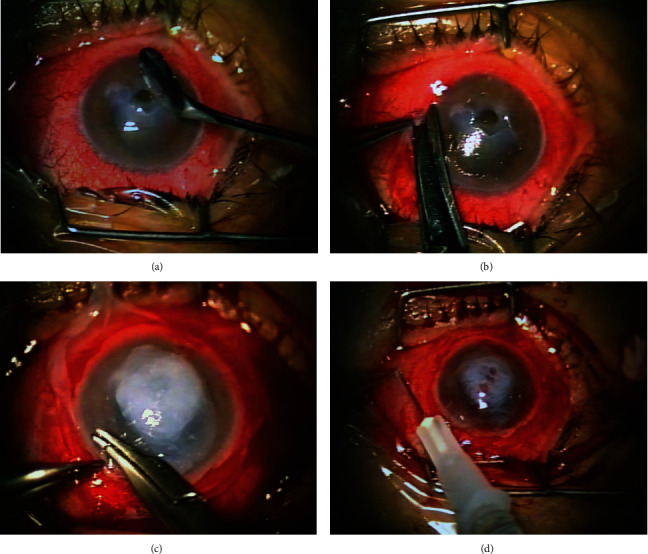
(a) The stromal ulceration was debrided, and the corneal epithelium was removed up to the limbal area. (b) 360° conjunctival peritomy is performed adjacent to limbus. (c) The ulcer was filled up to a surface level with multiple pieces of AM, and a larger AM was transferred to the recipient eye with the epithelial side down. The membrane was trimmed to cover the cornea and limbal area and secured with continuous 10-0 nylon sutures to the conjunctival edges. (d) After suturing, we do a subconjunctival injection with a mixture of dexamethasone and gentamycin. Finally, we place a bandage contact lens to cover the transplanted membrane and cornea.

Following peribulbar and subconjunctival anaesthesia, we carefully debrided the thinner crater and scraped off the corneal epithelium up to the limbal margin followed by a 360° conjunctival peritomy (Figures 1(a) and 1(b)). Small cut pieces of AM were used to fill the stroma defect level with the surface, and a larger piece was trimmed to cover the whole corneal and limbal area with the epithelial side down used (“sticky” stroma side up) as a patch for a healthy corneal epithelium to grow underneath. We secured the membrane under the conjunctival edges with a continuous 10.0 nylon suture (Figure 1(c)). Finally, we applied a subconjunctival injection of a mixture of dexamethasone and gentamycin and placed a bandage contact lens for 2 to 3 weeks (Figure 1(d)). Our routine postoperative regimen was combined steroid and antibiotic eye drops 6 times daily for two weeks tapering down thereafter.

For deep corneal ulceration, we use small pieces of AM to fill the stroma defect placed as grafts with epithelial side up aiming at subepithelial or transepithelial integration of the membrane in relation to the newly formed epithelium. Following limbal peritomy, a large piece of AM is placed as a patch with the epithelial side down covering the entire cornea and limbal area, aiming at superficial localization (disintegration) of this covering piece of amniotic membrane. In theory, this arrangement will facilitate the growth of a new, healthy corneal epithelium from the intact limbal area under the superficial layer of AM but over the small pieces of AM covering the stroma defect [3, 4]. We have abandoned the technique of using a smaller than the corneal diameter piece of AM as a graft (epithelium up) which is sutured on to the cornea within the limbus so that the newly formed epithelium will grow over the membrane as less effective and more traumatic for the cornea [5].

**Figure 2:**
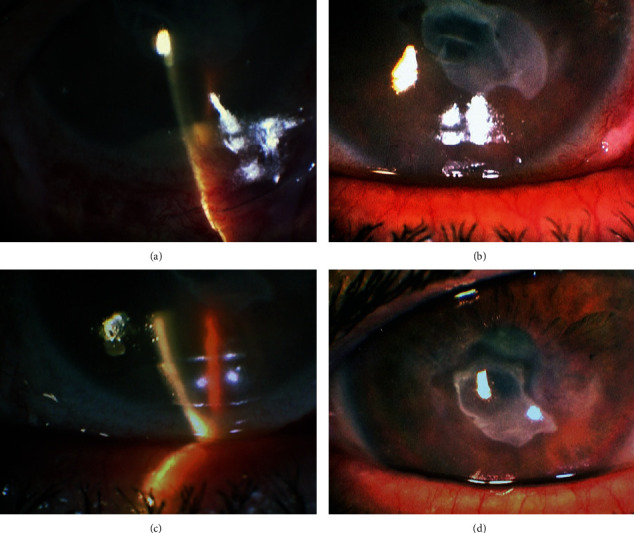
(a) 3 mm hypopyon formation 2 days after cryopreserved AMT. (b) Regression of hypopyon one week later. (c, d) Clear anterior chamber with healed ocular surface and no signs of inflammation 5 weeks after surgery.

The AM graft was prepared and cryopreserved by our local eye bank using the Good Tissue Banking Practice procedures [6]. Despite the tested sterility of the ocular surface, the preoperative antibiotic treatment, and the uneventful perioperative period, he developed a 2 mm hypopyon within 48 hours when we reviewed him after primary AMT (Figure 2(a)). He was using our routine regimen of tobramycin 3 mg/ml and dexamethasone 1 mg/ml eye drops six times daily on a fixed combination.

Despite the alarming clinical presentation, there were no anterior chamber reaction and no signs of active infection; therefore, we considered the hypopyon formation being a sterile toxic or immunologic reaction. To confirm this diagnosis, we took a surface swab from the membrane and performed a full microbiological examination of the residual membrane and its culture medium which we always keep for up to 7 days for quality control which were proven negative.

The patient continued with our standard post-AMT treatment with increased dosage every two hours for three days, followed by 6 times daily for two more weeks tapering down thereafter. The hypopyon resolved completely with the current treatment a week later with no further complications or anterior chamber reaction (Figure 2(b)).

The epithelial defect and corneal thinning healed satisfactorily with smooth epithelium and a visible stromal scar with no recurrence or hypopyon 5 weeks postoperatively (Figures 2(c) and 2(d)).

## 3. Discussion

We have previously described in detail the standardized method for AM preparation used in our local eye bank since 1999 [2]. The placenta is obtained from an uncomplicated elective caesarean section delivery following all the necessary serologic and microbiology tests for the donor and tissue. After cleaning and rinsing the membrane with antibiotics under sterile conditions (lamellar flow hood), we place it onto a sterile nitrocellulose paper with the epithelial side up which we cut into 3 × 4 cm pieces. These are placed in sterile vials containing Dulbecco's modified Eagle medium and glycerol in a ratio of 1 : 1 for deep freeze (-80°C) storage. The AM is defrosted immediately before surgery and washed in sterile saline at least three times to remove the storage solution completely The process follows all the tissue testing and sterility protocols with quality control and release criteria of tissue adhered to the Good Tissue Practice set by the FDA [6].

Two to five of the stored membranes from each placenta are randomly tested for infection before released for surgery to ensure sterility of the processing and storage method. Similarly, we retain redundant tissue and culture medium after surgery for up to one week for postoperative repeat testing when necessary as it happened in this case.

Infection and toxic or immunologic reaction are the three most possible pathophysiological mechanisms for hypopyon formation in the immediate postoperative period following AMT. Neurotrophic keratopathy which is often requires AMT can present hypopyon in the natural course of the disease, but our case had uncompromised corneal sensation.

We have excluded the possibility of infection on this selected case based on negative microbiology testing and the clinical presentation with no infectious signs. Furthermore, ocular surface toxicity from chemicals may induce anterior chamber reaction but this seems very unlikely because we use a standardized time-tested surgical technique which includes intensive rinsing of the storage medium from the membrane with normal saline before transplantation, and all other cases of AMT had an unremarkable postoperative period.

Thus, we consider an immunologic reaction as the most probable cause for hypopyon formation in this case. There is evidence that the epithelial cells of AM do not express human leukocyte antigens A, B, C, or DR; therefore, AMT onto the ocular surface is considered to be immunologically inert [1, 5]. There is no published evidence of documented immunogenic reaction or graft rejections related to AMT for ocular surface reconstruction in humans although Howell et al. reported histologic evidence of host versus graft reaction after repeated transplantation of bovine AM into cattle with glycogenosis type II [7].

A retrospective review of cryopreserved AMT cases for noninfectious ocular surface reconstruction performed over 15-year period (2005-2015) identified a single case with adequate documentation and imaging out of 525 (0.19%) who developed sterile hypopyon after surgery.

A detailed literature search (Medline, PubMed, and Cochrane database) revealed three reports of sterile hypopyon formation soon after AMT. Gabler et al. reported hypopyon formation possibly due to immunologic reaction after repeated transplantation of amniotic membrane (AM) onto the corneal surface. The reaction occurred after the second and third cryopreserved AMT from the same donor for sterile deep trophic corneal ulceration [8]. Messmer reported two similar cases of hypopyon formation following cryopreserved AMT. However, both reported cases had a significant general medical history with an active atopic dermatitis on the first case in which the hypopyon occurred 4 weeks (rather than a few days) after combined superficial keratectomy, limbal allograft, and AMT in the context of limbal graft rejection. The second case suffered an acute leukaemia treated with full-body irradiation and bone marrow transplantation and developed hypopyon formation 2 days after repeated AMT although a penetrating keratoplasty had been performed between the AMTs [9]. In contrast to our uncomplicated case, both reports had the common feature of prior sensitization and immune reaction by either repeated procedures or allograft tissue. In a similar report, Srinivasan et al. used fresh AM and considered the immunogenicity of the fresh unprocessed tissue as the cause for hypopyon formation [10]. It has been shown that cryopreservation in 50% glycerol preserves the structure of the epithelium of the amniotic membrane but devitalizes cells contributing to reduced immunogenicity compared with fresh membranes which may induce immunologic reaction and explain hypopyon iritis in this case. However, cryopreservation does not exclude the possibility of immune reaction to the remaining antigenic structures [1, 11].

We have used amniotic membrane preparation and surgical techniques comparable to the ones reported in these publications (Figures 1(a)–1(d)) [2]. The presented case developed sterile hypopyon within two days after primary cryopreserved AMT for sterile corneal ulceration with no prior immunological sensitization or other ocular surface disease and resolved with our routine topical treatment for AMT. This report highlights the fact that cryopreserved amniotic membrane, although immunologically inert, when transplanted onto the corneal surface may elicit a localized sterile immune reaction presenting with a transient hypopyon formation. There is a strong need for more investigations into the immunologic implications of AMT and possible complications related to AMT. The uncommon complication of hypopyon formation soon after AMT should be clearly differentiated from surface infection or even endophthalmitis and treated accordingly. The literature suggests that a particular care must be taken for cases of repeated AMT or combined use of allogenic tissue that may stimulate the ocular immune system and more importantly in patients with systemic immunologic disorders.

## Data Availability

The figures used to support the findings of this study are included within the article.
